# Prevalence and associated factors of COVID-19 among Moroccan physicians: A cross-sectional study

**DOI:** 10.1371/journal.pone.0277157

**Published:** 2022-11-02

**Authors:** Marwa El Baldi, Amina Laghrissi, Zakia Marso, Fatima Zahra Chellat, Mohamed Berraho, Nabil Tachfouti, Samira El Fakir, Soufiane Mellas, Amar Mohamed Fahd, Jamal kohen, Fouad Boulaguige, Jamal Naamane, Khalid Lahmadi, Karima El Rhazi

**Affiliations:** 1 Faculty of Medicine and Pharmacy, Laboratory of Epidemiology, Clinical Research, and Community Health, Sidi Mohamed Ben Abdallah University, Fez, Morocco; 2 Regional Direction of Health, Fez Meknes Region, Morocco; 3 Regional Council for the Order of Physicians, Fez-Meknes Region, Morocco; 4 Hematology Department, Moulay Ismail Military Hospital, Meknes, Morocco; Centre de Recherche Scientifique et Technique sur les Regions Arides, ALGERIA

## Abstract

**Background:**

Coronavirus disease (COVID-19) has emerged and spread rapidly worldwide and established a global public health crisis in early 2020. The first Moroccan case was reported on March 2, 2020. Since then, healthcare workers (HCWs) played a major role in saving human lives threatened by COVID-19. This study aimed to assess the prevalence of COVID-19 infection among Moroccan physicians and to report associated risk factors prior vaccination campaign.

**Methods:**

A cross-sectional study was carried out in the Fez-Meknes region of Morocco, 545 physicians’ data was collected using a self-reported online questionnaire. The data collection was done between December 1, 2020, and February 1, 2021.

**Results:**

The prevalence of COVID-19 among physicians was 27.3%. The mean age of the confirmed COVID-19 group was 38.4±12.9 years old. There was no association between COVID-19 infection and preventive measures compliance by physicians and healthcare authorities in the workplace. However, multivariate analysis strengthened the following factors such as increased risk of COVID-19 infection within men ☯aOR:1.896; 95% IC 1.272–2.828; p = 0.002]; the presence of at least one comorbidity ☯aOR:2.268; 95%IC 1.414–3.637; p = 0.001]; and working at a university or military hospitals ☯aOR:2.578; 95%IC 1.667–3.989; p = 0.001].

**Conclusion:**

This study allows comparing COVID-19 prevalence among healthcare workers before and after vaccination programs. This should support better preparation strategy for any future pandemics with appropriate and increased awareness for men, carrying comorbidity, and working environment with high COVID-19 disease management.

## Introduction

Coronavirus disease (COVID-19) is instigating severe and acute respiratory syndrome, The first emergent coronavirus was first recognized in Wuhan, Hubei province, China, in December 2019 [1]. COVID-19 has spread rapidly worldwide, and reached other countries despite quarantine, social distancing (SD) measures, and initiated a global public health crisis. on January 31, 2020, the global pandemic was recognized by the World Health Organization (WHO) as a public health emergency of international concern. Hence, a global pandemic was declared on March 11, 2020 by the WHO [2].

The North African countries experienced COVID-19 between February and March 2020 [3–13]. Between March 1 and 2, 2020, Morocco reported their first COVID-19 cases [6]. Three weeks after, the Moroccan National Council of the Order of Physicians declared COVID-19 infection in 11 physicians and two died 1 week later [14].

Healthcare workers (HCWs) played a main role in saving human lives threatened by COVID-19. Therefore, they carried a higher risk of COVID-19 and psychological distress compared to the general population [15–19]. During the early stage of the pandemic statistics showed that 3.9% HCWs were affected by SARS-CoV-2 worldwide [20]. The highest prevalence of HCWs infection was reported in Europe (78.2%) [20] and USA (11.0%) [21] while the lowest prevalence was reported in China (4.4%) [22] and Africa (1.0%) [20]. China and Italy were the first countries to report HCWs deaths and physicians were the largest entity of HCWs that died (51.4%) [20]. Hence, WHO and Centers for Disease Control and Prevention (CDCP) published guidelines for HCWs on preventing and controlling COVID-19 on June 29, 2020 [23]. The Moroccan health authorities instigated higher infection prevention and control measures to decrease the spread of COVID-19 in healthcare facilities [24]. HCWs working in high-risk environments such as intensive care units, isolation wards, emergency rooms, operating rooms, and general medical wards are required to wear PPE and recommended to maintain rigorous hand, staff, and workplace hygiene [25].

Thus, several studies have been carried out in Morocco assessing the management strategies in healthcare environments including primary care, intensive care units, hemodialysis units, blood transfusion, and cancer centers [26–36]. Studies also included ethical and legal issues related to hydroxychloroquine treatment prescription for COVID-19 and COVID-19 disease recognition as work-related accident [14,37]. Other studies evaluated stress, Post-traumatic stress disorder (PTSD), depression, anxiety, burnout factors, eating disorders, and quality of life during the pandemic in HCWs [38–44]. Predictive variables for impaired mental health were also investigated within medical students since they underwent a major change in education and training during the pandemic [45–47]. In addition, symptoms related to "Long-COVID-19" as well as signs of headaches and skin allergic reactions caused by PPE were exanimated among HCWs [48–50]. However, there was not any published study assessing the prevalence of COVID-19 and its risk factors among physicians.

Before February 1st, 2021, and before initiating vaccination campaign in Morocco, even to many worldwide countries [51–57], it appeared that workplace hygiene, protection policies and the workers compliance, were key and effective COVID-19 prevention in healthcare environment. In this context, this cross-sectional study was started to assess the prevalence of COVID-19 and its associated factors according to hygiene and protection measures among physicians in the Fez-Meknes region of Morocco.

## Methods

### Study population and area

This cross-sectional study was carried out by the Laboratory of Epidemiology, Clinical Research and Community Health in collaboration with the Council of the Order of Physicians of the Fez-Meknes region between December 1, 2020, and February 1, 2021.

Fez-Meknes region is one of the twelve regions of Morocco established by the territorial division of 2015. It is located in the north center of Morocco [58]. According to the Ministry of Health and social protection, this region’s overall population is 4,484,111; it includes both urban and rural areas. The hospital network consists of 21 hospitals, with a total of 3346 existing beds and 3012 functioning beds. This health facilities consists of public health sector that is operating with 2233 general practitioners/specialists, 57 dental surgeons/pharmacists, 4,651 nurses/midwives, 330 administrators, and 555 technicians; besides the private sector is detaining 1357 general practitioners/specialists. As result; the population ratio consists of 1340 per hospital bed, 10576 per primary public care institution network, 1249 per public physician, and 963 per public nurse [59].

This study targeted all physicians in the Fez-Meknes region. The sample size was calculated using OpenEpi 3.0 software [60]. The minimum required number (n) was 342 participants using the following formula:

n=[DEFF∗Np(1−p)]/[(d2/Z21−α/2∗(N−1)+p∗(1−p)]

Where, n = sample size, DEFF = design effect, N = population size (3080), p = estimated proportion, q = 1-p, d = desired precision or absolute level of precision, Z is a constant = 1.96 for 95% Confidence interval.

### Data collection

The questionnaire comprised 37 close-ended questions. It was designed by the laboratory of Epidemiology, Clinical Research, and Community Health team; it was based on extensive bibliographic research, official manuals, protocols, and guidelines in national and international equivalent contexts. This would allow achieving COVID-19 diagnostic according to symptoms, PCR, CT chest scan, and antigenic results. In addition, preventive measures needed in healthcare settings are also included.

A pilot feasibility study was accomplished to test the questionnaire, it was done in the university hospital, physicians volunteered to participate and the final version was approved after solving reported comments including incomprehension and other difficulties etc.

A Google form self-administered questionnaire was designed and distributed to all physicians of Fez Meknes Region via social media network including Facebook, WhatsApp, and e-mails.

The questionnaire consisted of 4 parts. The first one allows evaluating participants socio-demographic characteristics including gender; birth date; working hospital such as private, public, University Hospital, and Army; and physician’s practice profile such as intern, resident, general practitioner, and specialist.

The second part allows exploring the clinical variables including comorbidities such as obesity, diabetes, cardiovascular disease, cancer, pregnancy, and any other chronic pre-existing pathological entity; the presence any acute respiratory infection (ARI), and the presence potential typical COVID-19 symptoms within the early stage of COVID-19 pandemic such as fever, cough, general weakness/fatigue, headache, myalgia, sore throat, coryza, dyspnea, anorexia/nausea/vomiting, diarrhea, and altered mental status.

The third part of the questionnaire included data on working environment such as dealing with patients’ samples taken for assessing COVID-19, direct contact with a suspected/confirmed case in family, professional and social circles. COVID-19 diagnostic was confirmed basing on the Polymerase Chain Reaction (PCR) test and/or Computed Tomography (CT) scan.

The last section of the questionnaire consisted of evaluating the compliance to the implemented preventive measures in the workplace which were recommended by the Moroccan health authorities such as achieving training program against COVID-19; Sanitation and Hygiene (WASH) materials and services using hydroalcoholic solution, soap, water; PPE in sufficient quantity and quality; isolation unit for COVID-19 patients and adjusting the workload of healthcare workers. Besides, the questionnaire included items describing hygiene and protection measures that have to be applied by physicians in the workplace such as hands cleaning before and after dealing with all patients, suspected patients, and patient environment and use of PPE.

### Variable’s definitions

#### COVID-19 variables

The COVID-19 variables were grouped in two categories: confirmed vs suspected/unconfirmed.

The definition of COVID-19 confirmed case was based on guide of the Moroccan health authorities [61], it consists of considering positive RT-PCR **OR** a suspected case with CT images suggestive of COVID-19 as evidence of confirming COVID-19 case. This definition constituted the rational of initiation of the available treatment protocol of cases in health facilities during this stage of the pandemic. Besides, the Moroccan health authorities [61] defined the suspected case as a person who achieved a direct contact with a confirmed case during the period of contagiousness or a cluster and who developed either sign of acute respiratory infection including cough, sore throat breathing difficulty; **OR** a fever above 38°C without any obvious etiology; **OR** with agnosia, anosmia, and dysphagia without obvious etiology [61]. Finally, each participant not full filling the COVID-19 definition above was considered as unconfirmed.

### Other variables

- Age groups were repartitioned into two categories, the first group enclosed cases below 30 years-old and the second included cases of 30 years-old and above. This choice was based on the sample’s median, and findings indicating that of COVID-19 risk is increased with the age 30s’ [62].
- The included profile of physicians was classified into two groups, the first one includes general practitioners and specialists, and the second include interns and residents, the major consideration of this repartition was the level of skills, professional experience and medical training. General practitioners and specialists have had already completed their medical training, while interns and residents are still heading to finish but were working full-time under the supervision of specialized training staff.
- Compliance to the implementation of preventive measures by healthcare authorities was reached once materials are washed and services such as availability hydroalcoholic solution/water and soap, and PPE were available in the workplace in sufficient quantity and quality. The availability of COVID-19 training program, COVID-19 isolation unit, and adaptation of HCWs number to workload were not included in this criteria
- Adherence to hygiene and protection measures by physicians was reached if they wash their hands before and after touching any patient or patient environment, and if they used always/usually PPE.

### Ethical issues

Data was gathered anonymously, and participants in the study voluntarily agreed to take part by clicking the box that indicated, "I agree to participate in this study." During data collection and analysis, data confidentiality was protected. This study was approved by the Fez University Hospital Ethics Committee.

### Statistical analysis

All collected data were checked and descriptive statistical analysis was performed. Qualitative variables were described as percentages and quantitative data was expressed by mean ± standard deviation. During the analysis, the studied population was divided into two groups, a confirmed group with COVID-19 and suspected group. Pearson chi-square test was used for categorical data, Fisher exact test if appropriate and the 2-sample Student t-test for the continuous variables. Variables with a P<0.05 were included in the multiple logistic regression model. A P<0.05 was used to determine statistical significance. All statistics were achieved using SPSS software v21.

## Results

All physicians in the Fez-Meknes Region were invited to participate to this study, and 545 physicians completed the survey. This included 50.4% (n = 273) females. The mean age of participating physicians was 37.8±13.4 years-old ranging from 21 to 77 years-old. Besides, 52.8% (n = 288) of participants were employed in public hospitals. Finally, the sample included 52.8% of specialists and general practitioners and 47.2% of interns and residents.

The prevalence of COVID-19 was estimated at 27.3% (n = 149). There was no significant difference between the average age of the confirmed group aged 38.4±12.9 and the unconfirmed/suspected group aged 37.5±13.6 with a p-value of 0.508. Regarding the physicians with at least one comorbidity, there was a significant difference in the percentage between the confirmed group (29.1%) and the unconfirmed/suspected group (18.0%) with a p-value of 0.005. Male predominance was noticed in the confirmed group with 61.5% (n = 91) and a p-value of 0.001. The COVID-19 infection was also associated to working in a COVID-19 units such as care units and assessment laboratories, that 65.8% was found in the confirmed group compared 54.5% in the unconfirmed/suspected group with a p-value of 0.018. There were more physicians working at university or military hospitals in the confirmed group with a rate of 37.6% compared to 23.0% found in the unconfirmed/suspected group with p-value of 0.002. Table 1 is reporting extensively the findings of both groups.

**Table 1:** Association between sociodemographic and clinical data with the infection of physicians with COVID-19.

	Total n = 545	Confirmed group n = 149	Unconfirmed / Suspected group n = 396	P-value
**Age, *mean±sd*** ***Range***	37.8±13.4 (21–77)	38.4±12.9 (24–74)	37.5±13.6 (21–77)	**0.508**
**Age categories, %(n)**				
**<30**	46.0(244)	42.4(61)	47.4(183)	**0.300**
**≥30**	54.0(286)	57.6(83)	52.6(203)	
**Sex, %(n)**				
**Female**	50.4 (273)	38.5(57)	54.8(216)	0.001*
**Male**	49.6 (269)	61.5(91)	45.2(178)	
**Hospital of work, %(n)**				0.002*
Public hospital	52.8(288)	47.7(71)	54.8(217)	
Private hospital	20.2(110)	14.8(22)	22.2(88)	
University/ Military hospital	27.0(147)	37.6(56)	23.0(91)	
**Profile of physician, %(n)**				
General/Specialist	52.8(288)	54.4 (81)	52.3(207)	0.663
Resident/ Interns	47.2(257)	45.6(68)	47.7(189)	
**Comorbidities, %(n)**				
**Yes**	21.0(144)	29.1(43)	18.0(71)	0.005*
**No**	79.0(429)	70.9(105)	82.0(324)	
**Working at a COVID-19 unit, %(n)**				
**Yes**	57.7(304)	65.8(98)	54.5(206)	0.018*
**No**	42.3(223)	34.2(51)	54.5(172)	

Most physicians (87.0%) confirmed the availability of WASH materials and services such as hydroalcoholic solution/water and soap. However, less than a half (46.1%) reported access to PPE with sufficient quantity and quality in their workplace. Besides, results did not show any difference between the confirmed and the unconfirmed/suspected groups depending of the availability of COVID-19 training program with respectively 84.6% and 77.5% with a p value of 0.070; WASH materials and services was satisfied for 86.3% compared to 87.2% respectively with p-value of 0.804. And PPE was available in sufficient quantity and quality in both groups with 50.3% and 44.6%, respectively with a p-value of 0.228. Also, 68.6% in the confirmed group declared the availability of COVID-19 isolation units in their workplace compared to 67.8% in the unconfirmed/suspected group with a p value of 0.891. Both groups announced that the number of healthcare workers was adequate to the workload with 61.1% in the confirmed group and 60.7% in the unconfirmed/suspected group and with a p value of 0.948. According to mentioned variable’s definitions above, 89.1% of physicians reported working in environment respecting the implemented preventive measures. However, there was no association between this variable and COVID-19 infection with p-value of 0.494. Table 2 is reporting extensively the findings of both groups.

**Table 2:** Association between COVID-19 infection and the implementation of the preventive measures in the workplace and the adherence to hygiene and protection measures.

	Total population n = 545	Confirmed group n = 149	Unconfirmed/suspected group n = 396	P-value
The availability of training program against COVID-19, %(n)	79.4(433/545)	84.6(126)	77.5(307)	0.070
The availability of WASH materials and services (Hydroalcoholic solution/water and soap), %(n)	87.0(354/407)	86.3(101)	87.2(253)	0.804
The availability of PPE with sufficient quantity and quality, %(n)	46.1(251/544)	50.3(75)	44.6(176)	0.228
The availability of COVID-19 isolation units, %(n)	68.0(219/322)	68.6(59)	67.8(160)	0.891
Adaptation of number of healthcare workers to the workload, %(n)	60.8(290/477)	61.0(83)	60.7(207)	0.948
**Compliance to the implementation of preventive measures by healthcare authorities**	89.1(450/505)	90.6(126)	88.5(324)	0.494
Cleaning hands before and after touching any patient or a suspected patient or patient environment, %(n)	98.8(501/507)	98.6(138)	98.9(363)	0.670
Using PPE, %(n)	89.9(473/544)	91.7(132)	89.3(341)	0.415
**Adherence to hygiene and protection measures by physicians**	39.6(216/545)	58.4(87)	61.1(242)	0.563

The hygiene and protection measures of physicians was not statistically significantly different in confirmed compared to unconfirmed/suspected groups according, this assessed hands cleaning before and after touching any patient and suspected patient and represented 98.6% versus 98.9% respectively in both groups with p-value of 0.670, while using PPE represented 91.7% and 89.3% respectively in both groups with p-value of 0.415. However, 39.6% of responding physicians were considered respecting the adherence criteria of hygiene and protection measures whenever applied. Thus, there wasn’t any association between this variables and COVID-19 infection with p-value of 0.563. Table 2 is reporting extensively the findings of both groups.

The unadjusted logistic regression revealed a higher risk of COVID-19 infection in men compared to women ☯OR:1.93, 95% CI 1.317–2.850]; besides, physicians without any comorbidity compared to those who had none showed ☯OR:1.869, 95%IC 1.206–2.896]. Physicians working in COVID-19 unit expressed ☯OR:1.604, 95%IC 1.082–2.380]; while physicians working in the university and military hospitals showed ☯OR:2.018, 95%IC 1.345–3.028]. Multivariate analysis strengthened the association between COVID-19infection and male gender with an ☯aOR:1.896, 95%IC 1.272–2.828, p = 0.002]; the presence of at least one comorbidity ☯aOR:2.268, 95%IC 1.414–3.637, p = 0.001] and working at the university or military hospitals ☯aOR:2.578, 95%IC 1.667–3.989, p<0.001]. Table 3 is reporting extensively the findings of both groups.

**Table 3:** Univariate and multivariate analysis between infection with COVID-19 with socio-demographic characteristics and adherence to hygiene and protection measures.

	Unadjusted Odds Ratio	95%IC	Adjusted Odds Ratio*	95%IC	P-value
**Age**					
≥30	1.227	0.834–1.805	-	-	-
<30	Ref		-	-	-
**Gender**					
Male	1.937	1.317–2.850	1.896	1.272–2.828	0.002
Female	Ref		Ref		
**Hospital (Other/University or military)**					
University or military	2.018	1.345–3.028	2.578	1.667–3.989	<0.001
Other	Ref		Ref		
**Comorbidities**					
Yes	1.869	1.206–2.896	2.268	1.414–3.637	0.001
No	Ref		Ref		
**Profile**					
interns and residents	0.919	0.630–1.342	-	-	-
Others	Ref		-	-	-
**Working at a COVID-19 unit (No/Yes)**					
Yes	1.604	1.082–2.380	-	-	-
No	Ref		-	-	-
**Compliance to the implementation of preventive measures by healthcare authorities**			-	-	-
Yes	0.796	0.413–1.533	-	-	-
No	Ref		-	-	-
**Adherence to the hygiene and protective measures by physicians**					
Yes	0.893	0.609–1.310	-	-	-
No	Ref		-	-	-

## Discussion

By the COVID-19 pandemic in Morocco, several studies have been carried out and covered the several issues that were considered challenging the health system [14,24,26–49,63]. However, there was inadequate data assessing the COVID-19 prevalence among Moroccan healthcare workers. To our knowledge, this cross-sectional is the first to assess the prevalence and associated factors of COVID-19 among physicians prior to any vaccination program. The results of this study demonstrated a COVID-19 prevalence of 27.3% among participants. The risk of COVID-19 infection was significantly higher in men; among physicians with at least one comorbidity, group of physicians working in COVID-19 unit, and physicians working at university or military hospitals.

Indeed, the rate of COVID-19 infection among physicians was higher than that in China (1.1%) [64], Italy (3.4%) [65], Oman (4.3%) [66], Bangladesh (10.7%) [67] and India (11.0%) [68]. However, lower than that found results in Brazil (42.3%) [69], UK (43.3%) [70], and Egypt (65.4%) [71]. The difference in reported rates of different countries depend of the sample size, the targeted medical personnel categories, the period of the study, and the screening protocols for COVID-19 infection in hospitals. Low routine screening of asymptomatic healthcare workers was the primary reason for the recorded COVID-19 infection rate in China, Italy, UK, and Oman [64–66,70]; in addition to low compliance with infection control measures, social distancing and generalized mask wearing during the pause. Bangladeshi authors confirmed that higher rate of infection might occur due to the non-use of appropriate PPE, inadequate training on the use of PPE, or the reuse of PPE [67], as well as two-thirds of the employees were not satisfied with the preventive measures undertaken by the health authorities. Besides, smoking, front-line workers, being in contact with a case of COVID-19, and working for less than 10 years were all factors associated with an increased risk of infection in Egypt [71].

We reported male predominance in the confirmed group (56.1%). This rate similar to reported studies in all HCWs such as found in Egypt (66.2%) [71] and Italy (75.0%) [65], while being higher compared to findings from Oman (9.3%) [66], UK (44.6%) [70] and China (71.8%) [64]. Previous data showed that males HCWs tended have less attention to the outbreak and recorded less anxiety and fear [72]; they expressed lower scores than women in effective personnel protection, including handwashing and wearing masks and gloves [72] and they showed higher rates of smoking [73] and alcohol drinking [74]. These factors were added to multiple findings such as difference in viral loads, antibody titers, plasma cytokines, and sex chromosome genes and hormones [75–77]. These might explain the reasons behind the high infection in male physicians compared to women.

Furthermore, preliminary evidences in the literature suggested an association between comorbidities such as chronic lung illness, hypertension, cardiovascular disease, diabetes, and rheumatism diseases; and the severity of COVID-19 [78–80]. In this study, 30.8% of confirmed physicians had at least one comorbidity, which is similar to Bangladesh result (29.7%) [67]. In contrast, this rate was higher compared to China (12.7%) [64]. Multiple data found that hypertension was the most common comorbidity among affected healthcare workers [64,81]. This finding can be explained by the use of hypertensive drugs that reduce blood pressure mostly through Angiotensin-converting enzyme (ACEi) and Angiotensin- receptor blockers (ARBS), which lead to increased ACE2 expression used by SARS-CoV-2 for human cell entry [82].

University and military hospitals were the most used hospital to care COVID-19 patients at the start of the pandemic. The staff of these hospitals expressed a higher COVID-19 infection rate. Additionally, a significant fraction of the confirmed group was recorded among interns and residents (44.1%) who were servicing in these hospitals. A similar result was reported by an Egyptian study which showed a high infection rate of residents (72.3%) [71]. This finding is explained by higher stress, long duty hours spent and deficiency in skills and experience. In fact, the national inter-union of interns (ISNIL) body conducted a study between May and July 2019 [83], and reported that interns spend an average of 58 hours/week/intern without rest. Evermore, they serviced up -to 80 hours and 8 to 10 on-call shifts each month. Another study showed that long duty hours were associated to COVID-19 infection risk [84]. Furthermore, residents and interns are expanding their newly acquired medical knowledge with the care of respiratory illnesses, which is still unknown for the COVID-19 infection and might expose them to higher infection risks.

COVID-19 may be transmitted through direct contact with COVID-19 patient; and indirect contact by touching the immediate environment of the infected person such as clothes, utensils, furniture, telephones, TV remote controls, countertops, etc. [85]. Researchers in Cleveland showed that 25% of positive COVID-19 healthcare workers expressed a higher risk of exposure at work, including 18% exposure to suspected patients and 6% to infected personnel [86].

In this study, the risk of infection was higher among physicians who worked in COVID-19 units including care units and laboratories. Similarly, a prospective study carried out in UK and USA showed that, HCWs involved in COVID-19 patients care were found to be five times higher a risk than working in general patient care despite using PPE [87]. Additionally, a Denmark observational cohort showed a higher seroprevalence among healthcare workers working in dedicated COVID-19 wards compared to those working in other units [88]. Other studies showed that, close patient contact, inappropriate microbiological techniques, unused PPE, lack of training, and inadequate decontamination protocols, are all exposing HCWs and laboratory workers to a higher risk of COVID-19 infection [89,90].

However, the COVID-19 transmission could be reduced by controlling indoor dust level, temperature, humidity, ventilation, improved hygiene, sanitization, wearing a mask and using PPE by HCWs [91]. Hence, Moroccan healthcare authorities have supported the major measures allowing to protect HCWs while following the most preventive guidelines published by WHO and CDC [21].

The undertaken measures include an implemented efficient educational training program in healthcare facilities, this allowed ensuring the support of the adequate practices during the pandemic waves. Also, it allowed increasing knowledge about all aspects of COVID-19 disease, ranging from symptomatology and diagnosis to treatment and prevention strategies. In this study, most participants (79.4%) received training for COVID-19 in their institutions. This training finding was higher compared to Southern Ethiopia (36.1%) [92] and Yemen (41.0%) [93]. Second, the access to water, sanitation, and hygiene (WASH) services was a key requirement of quality care and infection prevention and control in healthcare facilities (HCFs), As a result, the most of physicians (87.0%) confirmed that WASH services and hygiene materials were accessible in their workplaces including hydroalcoholic solutions, soap, and sufficient water for hand-washing.

Third, to protect HCWs sand other patients, CDC recommended to setup a triage protocols in hospital admission services to better care any acute respiratory symptoms (ARIS) [94]. Any patient arriving to hospitals’ facilities are provided and recommended permanent face masks dress-in. Besides, HCWs should were fully equipped with PPE. Suspected COVID-19 patients were isolated safely and rapidly. Therefore, more than half of reporting physicians (68.0%) confirmed the availability of COVID-19 isolation units in their workplace.

Finally, overworking and lack of rest indirectly increased the risk of infection and mortality among HCWs [95]. Hence, healthcare authorities improved human resources management by adapting restricting the number of workers per workload and reducing the work intensity. Most reporting physicians in this study (60.8%) indicated that the number of HCWs in their facilities was adequate to the workload. Indeed, the participating physicians have demonstrated a great responsibility towards respecting the preventive measures implemented in their working environments. Most physicians (98.8%) were compliant to measures such as cleaning hands using alcohol-based hand rubs, washing hands properly with soap and water, and using other antiseptic agents before and after touching any patient. This result was similar to Nigeria finding that consisted of up-to (96.0%) [96]; However, a Chinese study showed that 85.7% of COVID-19 positive HCWs had deficient hand washing, 78.57% and 60.71% demonstrated lower hygiene before and after contact with patients respectively [64]. Additionally, most participants (89.9%) used PPE contrast to Southern Ethiopia physicians who did not use masks despite their availability at the workplace [92].

These findings indicated that the majority of physicians and healthcare authorities were well complying to all workplace hygiene and protection guidelines advised by the relevant health organizations to prevent against COVID-19. However, there was no association between COVID-19 infection and any of these hygiene and protection measures practiced. In fact, being male sex, the presence of at least one comorbidity, and working at university or military hospitals were the found to be the major risk factors. Thus, the availability protective equipment in healthcare facilities, the ongoing commitment to hand hygiene, and the use of PPE were not sufficient to protect physicians in the most effective way against COVID-19 infections.

Our findings are not projected to issue recommendations but they could have a major interest to optimize working protocols for the decision-makers, organizations issuing recommendations, directors of health institutions and other employers, researchers, occupational health stakeholders. These results could be also useful to reinforce the protection measures and hygiene in COVID-19 units including laboratories and care units, with additional attention should be paid to men, physicians with at least one comorbidity, and personnel working at high workload hospitals.

Despite reported results and conclusion, limitations of this study should be noticed. This is a cross-sectional study achieved during the first wave of COVID-19 of the pandemic. The findings are self-reported and relatively depending on participants’ psychology, which is important during such difficult period. Thus, results are subjected to recall bias. The participants’ selection was based on the willingness to access to social networking platforms. Among all invited physicians, 545 responded to the questionnaire; this could be explained by a physicians’ lack of use of social platforms, particularly among elderly portion of physicians. Another limitation is related to the studied preventive measures that worked before the Omicron COVID-19 variant wave spread that has different characteristics and it is probably not functioning today and therefore the results should be carefully interpreted. However, this study was carried out in the regional context, the number of participants was equivalent to previous studies reporting all HCWs categories. Our findings provided valuable information about the prevalence of COVID-19 and associated risk factors among physicians during a peak period of COVID-19 and prior to any vaccination program.

## Conclusion

Healthcare workers continue running a high risk to be infected COVID-19, while the long-term vaccine efficiency has not been established. This study is helpful for comparing COVID-19 disease prevalence among HCWs before and after vaccination. Besides, it allows better preparing for any future pandemics, with increased vigilance for men, HCWs with at least one comorbidity, and HCWs working in hospitals with high COVID-19 disease supervision.
